# Syndrome d’Ogilvie, une complication rare de la chirurgie du canal lombaire étroit: à propos de deux cas et revue de la littérature

**DOI:** 10.11604/pamj.2022.42.2.21183

**Published:** 2022-05-04

**Authors:** Gbètoho Fortuné Gankpé, Laurent Do, Mohammed Rabhi

**Affiliations:** 1 Service de Neurochirurgie, Centre Hospitalier Universitaire Hassan II, Fès, Maroc,; 2 Service de Neurochirurgie, Centre Hospitalier Universitaire, Guadeloupe, France

**Keywords:** Syndrome d’Ogilvie, canal lombaire étroit, scanner abdominal, cas clinique, Ogilvie´s syndrome, lumbar stenosis, abdominal CT scan, case report

## Abstract

Le syndrome d´Ogilvie est une pseudo-occlusion colique aiguë, caractérisée par une distension colique avec risque de perforation caecale en absence de tout obstacle mécanique. C´est une pathologie très rare après une chirurgie rachidienne. Nous rapportons deux cas enregistrés dans le service de neurochirurgie du CHU de Guadeloupe. Il s´agit d´une femme de 79 ans en surpoids (IMC=27kg/m^2^) et un homme de 56 ans présentant des lombosciatagies bilatérales mal systématisées avec réduction du périmètre de marche évoluant depuis plusieurs mois chez qui l´IRM lombaire avait révélé un canal lombaire étroit et hernie discale, avaient subi une intervention chirurgicale de décompression par laminectomie lombaire. Ils ont présenté une constipation avec arrêt des matières et gaz 48h après la chirurgie et un ballonnement abdominal. Le scanner abdominal et la radiographie de l´abdomen ont montré une importante distension intestinale sans obstacle faisant évoquer un syndrome d´Ogilvie. Un traitement conservateur avait été suffisant pour traiter ce syndrome et les patients avaient complètement récupéré. Dans la survenue du syndrome d´Ogilvie, la pathologie la plus fréquente pour laquelle le geste chirurgical rachidien est en cause, est la hernie discale lombaire. La présentation clinique est classique avec un arrêt des matières et des gaz, un météorisme abdominal témoignant d´une distension intestinale volumineuse. Le traitement médical conservatoire reste le traitement de choix lorsque le diagnostic est fait tôt.

## Abstract (en)

Ogilvie´s syndrome is an acute colonic pseudo-obstruction, characterized by massive colonic distension in the absence of mechanical cause. It is a very rare pathology after spinal surgery. We report two cases in the neurosurgery department of the University Hospital of Guadeloupe. A 79-year-old woman overweight (BMI= 27kg/m^2^) and a 56-year-old man experienced history of non-systematized bilateral lumbar and sciatic pain with reduction in walking perimeter for few months. MRI of lumbar spine had revealed a lumbar stenosis with disc herniation. They had undergone decompression surgery with laminectomy. The surgical intervention was uneventful perioperatively. By 48 hours after surgery, they had complained of constipation with cessation of fecal and flatus with resultant abdominal distension. Abdominal CT scan and X-rays showed significant bowel distension with no mechanical obstruction, suggestive of Ogilvie´s syndrome. Conservative treatment had been sufficient to treat this syndrome and the patients completely recovered. In the occurrence of Ogilvie´s syndrome, the most frequent pathology is the lumbar disc herniation. The clinical presentation is typical with a cessation of fecal and gas elimination, and abdominal distension. Conservative treatment remains the treatment of choice when diagnosis is made early.

## Introduction

Le syndrome d´Ogilvie est une pseudo-occlusion colique aiguë, caractérisée par une distension colique avec risque de perforation caecale en absence de tout obstacle mécanique chez certains patients prédisposés du fait de leur pathologie, antécédents ou chirurgie [1]. William Ogilvie (1887-1971) fut le premier à décrire cette entité en 1948 chez deux patients porteurs de cancers rétropéritonéaux ayant envahi le plexus cœliaque faisant évoquer une privation des influences sympathiques comme étiologie [2]. La physiopathologie a beaucoup évolué et le mécanisme admis à nos jours serait un dysfonctionnement du système parasympathique. Il s´agit d´une pathologie rencontrée au décours d´une chirurgie notamment la chirurgie digestive. En revanche, de très rares cas ont été rapportés en chirurgie du rachis. Nous rapportons ici deux cas observés chez des patients opérés pour un canal lombaire étroit.

## Patient et observation

la première patiente était une femme de 79 ans, retraitée, ayant comme antécédents un diabète de type 2, une hypertension artérielle bien suivie, sous amlodipine, césarienne et surpoids avec un indice de masse corporelle (IMC) = 27kg/m^2^, présentant depuis plusieurs années des lombosciatalgies bilatérales mal systématisées prédominant à droite avec réduction du périmètre de marche. Le deuxième patient était un homme de 56 ans, facteur de profession, portant donc régulièrement des charges, ayant comme antécédent une hypertension artérielle sous amlodipine, suivi pour un canal lombaire étroit évoluant depuis 2 mois environs, qui avait présenté à la suite d´un port de charge lourde, des lombalgies intenses.

**Examen clinique**: la patiente présentait des douleurs rachidiennes lombaires et une raideur du rachis lombaire. Elle n´avait pas de déficit sensitivo-moteur. Le patient de 56 ans présentait un déficit radiculaire L5 gauche obligeant le patient à marcher à l´aide d´une canne.

**Démarche diagnostique**: l´IRM lombaire de la patiente de 79 ans avait objectivé un canal lombaire étroit. L´IRM lombaire réalisée chez le patient de 56 ans avait révélé une hernie discale L4-L5 paramédiane gauche décompensant un canal lombaire étroit. L´indication neurochirurgicale avait été retenue chez les deux patients.

**Intervention thérapeutique**: la chirurgie avait consisté à une laminectomie à 3 étages L3, L4 et L5 chez les deux patients. Elle était complétée par une discectomie L4-L5 gauche chez le patient de 56 ans.

**Figure 1:**
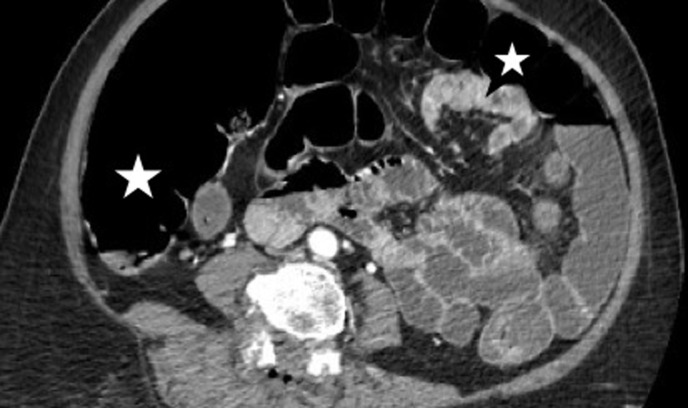
scanner abdominal en coupe axiale de la patiente 1 montrant une distension intestinale, sans signe de perforation caecale de la patiente (astérisque)

**Figure 2:**
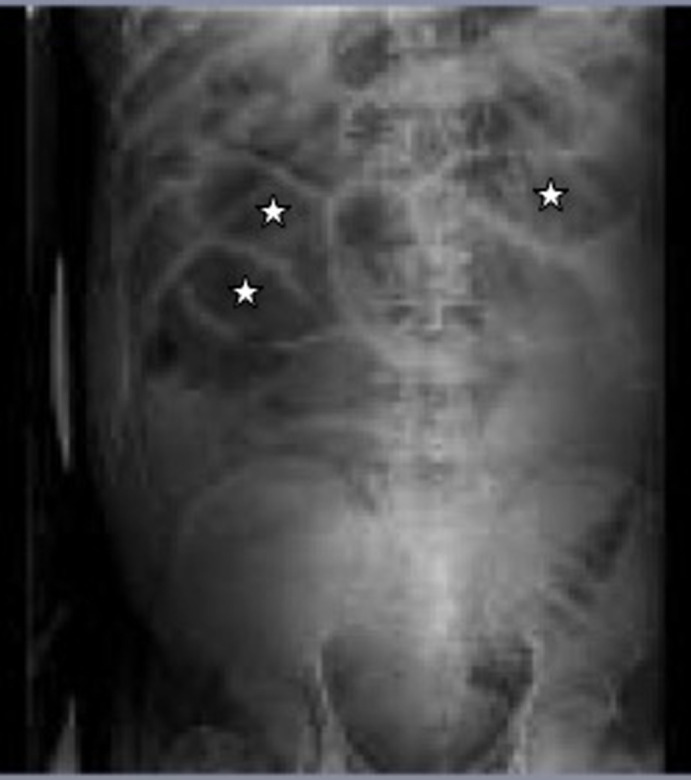
radiographie de l'abdomen sans préparation du 2^e^ patient montrant des aérocolies (astérisque) sans signe de perforation

**Suivi**: chez la patiente de 79 ans, les suites post-opératoires étaient marquées par la survenue d´une constipation avec arrêt des matières et des gaz 3 jours plus tard, puis une détresse respiratoire avec un syndrome subocclusif franc. Devant la menace vitale immédiate, elle avait bénéficié de mesures de réanimation (aspiration nasotrachéale, oxygénothérapie), et aussi d´une évacuation gastrointestinale en amont et en aval avec une sonde nasogastrique en aspiration à demeure et des laxatifs osmotiques. Le scanner abdominopelvien (Figure 1) avait révélé une distension abdominale importante sans obstacle et sans perforation caecale faisant évoquer un syndrome d´Ogilvie post-opératoire. Sous le traitement médical entrepris, l´évolution était favorable et la patiente était mise en exéat au 9^e^ jour d´hospitalisation. Le contrôle radioclinique à 3 et 6 mois était sans particularité. Le patient de 56 ans avait présenté dans les suites opératoires, une constipation avec arrêt des matières et des gaz, un ballonnement abdominal franc avec défense abdominale. La radiographie de l´abdomen sans préparation avait révélé une distension intestinale (Figure 2) et un doute sur un obstacle motivant la réalisation d´une radiographie avec préparation à la gastrografine sans obstacle sur le transit, faisant évoquer un syndrome d´Ogilvie post-opératoire. Il n´y avait pas de signe de perforation caecale. Le patient avait alors bénéficié d´une évacuation gastrointestinale en amont et en aval avec des laxatifs osmotiques et une sonde nasogastrique en aspiration à demeure. L´évolution était favorable et le patient était sorti au 8^e^ jour post-opératoire. Le contrôle radioclinique à 3 et 6 mois était sans particularité. Les deux patients étaient opérés le même jour. La position opératoire était le décubitus ventral, avec un billot sous l´abdomen; le temps opératoire était raisonnable d´une heure et trente minutes. On notait un usage d´analgésique à des doses maximales comprenant la morphine à la dose de 10mg/Kg toutes les 4h. Il n´y avait pas d´accident per-opératoire, ni anesthésique.

## Discussion

Le syndrome d´Ogilvie tel que décrit par Heneage William Ogilvie lui-même est une pseudo-occlusion présentant les mêmes caractéristiques cliniques qu´un syndrome occlusif franc en dehors de tout obstacle sur le transit intestinal. Il s´agit d´un trouble de la motilité intestinale décrit comme une distension colique massive en absence de tout obstacle mécanique [2]. La physiopathologie est encore mal connue. On sait que la motilité colique et les fonctions sécrétoires du tube digestif sont médiées par le système nerveux autonome sympathique et parasympathique. Les fibres du système nerveux sympathique proviennent de la moelle épinière thoracique et lombaire et celles du système nerveux parasympathique proviennent du nerf vague et les racines spinales sacrées de S2 à S4 [1]. Ogilvie partait de trois hypothèses physiopathologiques pour finalement retenir le mécanisme d´une interruption des influences sympathiques dans ces deux cas publiés, ce qui lève donc l´inhibition de la voie parasympathique qui se trouve renforcée [2]. En revanche, les résultats satisfaisants obtenus par l´utilisation de la néostigmine qui est un agent stimulant les influences parasympathiques ont permis d´évoquer la théorie de la privation nerveuse parasympathique dans la genèse du syndrome d´Ogilvie [3].

Depuis la description des deux cas d´Ogilvie, plusieurs études ont rapporté les facteurs de risque exposant à la survenue du syndrome d´Ogilvie. Dans la série de Vanek sur 400 cas, le sexe masculin était le plus représenté avec une moyenne d´âge de 60 ans; 94,5% des cas étaient associés à d´autres pathologies médicales ou chirurgicales [4]. Tenofsky et collaborateurs avaient noté une moyenne d´âge de 68,9 ans avec un sex ratio de 2/1 (2: 1) en faveur des hommes [3]. Nos deux patients avaient respectivement 79 et 56 ans, une femme et un homme. Les pathologies en cause les plus souvent rapportées dans la littérature sont: les traumatismes liés aux accidents de la voie publique, les infections, les cardiopathies, les insuffisances rénales, le diabète, et les troubles métaboliques [1,3,5-7].

Les facteurs favorisant un syndrome d´Ogilvie retrouvés dans la littérature sont: l´obésité, l´usage de drogues analgésiques fortes, les narcotiques, un antécédent de césarienne, l´âge des patients, l´usage d´inhibiteurs calciques notamment la nimodipine [1,3,4,6,7]. Dans notre série, on notait l´hypertension artérielle traitée par des inhibiteurs calciques, le surpoids, le diabète, les analgésiques utilisés en période péri-opératoire, la césarienne. Les suites opératoires représentent selon certains auteurs 50 à 60% des cas de syndrome d´Ogilvie rapportés dans la littérature [1,3,6,7]. Cependant très peu d´auteurs ont mis un accent particulier sur la survenue d´un syndrome d´Ogilvie suite à une chirurgie rachidienne. Notre revue a permis de retrouver au total 9 cas rapportés dans la littérature (Tableau 1).

**Tableau 1:** cas de syndrome d´Ogilvie après une chirurgie rachidienne rapportés dans la littérature

Auteurs	Cas	Age	Sexe	Pathologie	Intervention chirurgicale	Traitement
**Çakir *et al*., 2001 0000000 [1]**	5	42	M	Hernie discale C5C6	Discectomie + arthrodèse	Conservateur, néostigmine
**Caner *et al*., 2000 [2]**	4	43	F	Hernie discale L4L5	Discectomie L4L5	Conservateur
**Feldman R & Karl R, 1992 [3]**	1	62	M	CLE + HD L2L3	Laminectomie L1L5 + Discectomie L2L3	Laparotomie & caecostomie
	2	58	M	CLE+ Spondylolisthesis L4L5	Laminectomie + fusion L4L5	Laparotomie + caecostomie
	3	42	M	Hernie discale L4L5	Discectomie L4L5	Colonoscopie
**Reverdy D *et al*., 2006 [6]**	9	79	M	Métastase vertébrale L3	Ostétomie + ostéosynthèse	Conservateur, néostigmine puis laparotomie + hemicolectomie droite
**Vega-Basulto *et al*., 2002 [8]**	6	36	M	Hernie discale L4L5	Discectomie L4L5	Conservateur, néostigmine
	7	41	M	Hernie discale L4L5	Discectomie L4L5	Conservateur, néostigmine
	8	42	M	Hernie discale L5S1	Discectomie L5S1	Conservateur, néostigmine

On note une nette prédominance du sexe masculin, 8 cas sur 9 et la tranche d´âge variait de 36 à 79 ans. La pathologie la plus fréquente était la hernie discale lombaire et l´intervention chirurgicale était en conséquence une discectomie. Il n´y a pas de facteurs spécifiques à la chirurgie du rachis, prédisposant au syndrome d´Ogilvie. Certains auteurs avaient évoqué le rôle d´un hématome rétropéritonéal post-opératoire, la position opératoire notamment le décubitus ventral qui exercerait une certaine pression sur l´abdomen, la durée de l´intervention chirurgicale, et l´usage de drogues opioïdes souvent utilisés chez ces patients [5,6,8,9]. Dans notre série, en particulier pour le premier cas, la possible compression abdominale exercée par la position en décubitus ventral chez une patiente en surpoids, sur un billot assez peu adapté, aurait probablement joué un rôle dans la survenue du syndrome d´Ogilvie. Nous souscrivons à la théorie de l´interruption des influences parasympatiques dans la génèse du syndrome d´Ogilvie au regard du facteur prépondérant de la compression abdominale et l´usage à des doses maximales d´analgésiques dans notre série, nos patients n´ayant en commun que ces facteurs.

**Figure 3:**
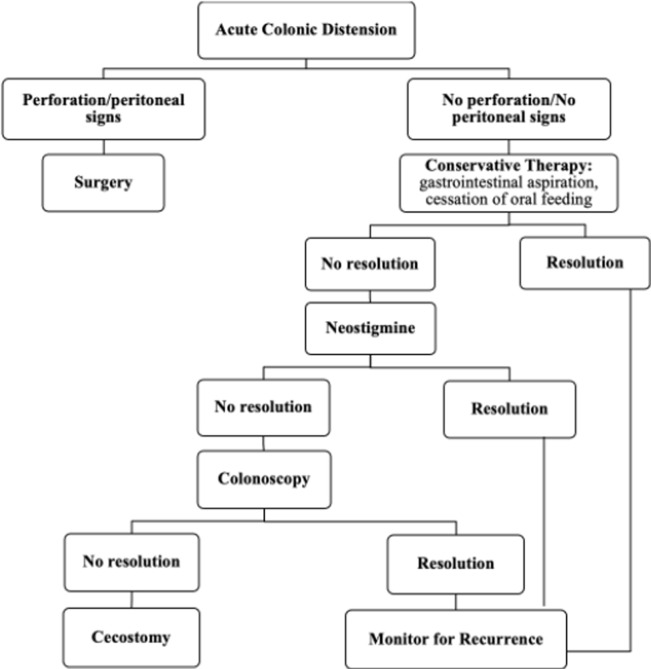
algorithme de la démarche thérapeutique du syndrome d'Ogilvie selon Maloney et Vargas [4]

Le traitement conservateur semble donner de bons résultats. Il est basé sur l´aspiration gastro-intestinale, un arrêt de l´alimentation par voie orale jusqu´à rétablissement du transit intestinal, et l´usage de la néostigmine quelques fois. Une laparotomie avec colectomie ou caecostomie était nécessaire dans 3 cas dont un cas après l´échec du traitement conservateur; la colonoscopie était utilisée dans 2 cas. Le débat sur la place du traitement chirurgical d´emblée est encore d´actualité. L´indication chirurgicale d´une laparotomie est fonction du diamètre de la distension caecale et de l´état clinique du patient. Il n´y a pas de consensus sur le seuil indicatif, il varie entre 9cm ou 12cm de diamètre selon les auteurs [1,6,7]. Maloney et Vargas, 2005 ont proposé un algorithme sur la démarche thérapeutique. Globalement, le traitement conservateur est indiqué en première intention, et la chirurgie intervient en cas d´échec (Figure 3) [4]. Les deux patients de notre série, conformément à l´algorithme ci-dessous ont été pris en charge de façon conservatrice avec une bonne récupération complète sans recours à la néostigmine.

## Conclusion

Le syndrome d´Ogilvie reste une cause très rare de complications après une chirurgie du rachis. La présentation clinique est classique avec un arrêt des matières et des gaz, un météorisme abdominal témoignant d´une distension intestinale volumineuse. La radiographie de l´abdomen sans préparation et le scanner permettent de poser le diagnostic en révélant la distension caecale sans obstacle mécanique. Une perforation caecale conduisant à une péritonite est la complication majeure. Le traitement médical conservateur reste le traitement de choix lorsque le diagnostic est fait précocement.
